# Leprosy with type 1 reaction in a patient from Ontario, Canada without recent travel misdiagnosed as vasculitic neuropathy: a case report

**DOI:** 10.1186/s12879-023-08811-x

**Published:** 2023-11-21

**Authors:** Matt Driedger, Iris Teo, Virginia Roth

**Affiliations:** 1 University of Ottawa, Ottawa, ON Canada; 2 Division of Anatomical Pathology, The Ottawa Hospital, Ottawa, ON Canada; 3 The Ottawa Hospital, Ottawa, ON Canada; 4 The Ottawa Hospital Research Institute, Ottawa, ON Canada

**Keywords:** Mycobacterium leprae, Leprosy, Reaction, Ontario, Neuropathy

## Abstract

**Background:**

Leprosy is rare within non-endemic countries such as Canada, where cases are almost exclusively imported from endemic regions, often presenting after an incubation period of as many as 20 years. Due to its rarity and prolonged incubation period, diagnosis is often delayed, which may result in neurologic impairment prior to the initiation of treatment. In this report we describe a case that is novel in its incubation period, which is the longest reported to-date and may have contributed to diagnostic delay. The case also uniquely demonstrates the challenges of distinguishing leprosy reactions from new rheumatologic manifestations in a patient with established autoimmune disease.

**Case presentation:**

We describe an 84-year-old male patient with rheumatoid arthritis on methotrexate and hydroxychloroquine, with no travel history outside Canada for 56 years, who presented in 2019 with new-onset paresthesias and rash. His paresthesias persisted despite a short course of prednisone, and his rash recurred after initial improvement. He underwent skin biopsy in May 2021, which eventually led to the diagnosis of leprosy. He was diagnosed with type 1 reaction and was started on rifampin, dapsone, clofazimine and prednisone, with which his rash resolved but his neurologic impairment remained.

**Conclusion:**

This case report serves to highlight the potential for leprosy to present after markedly prolonged incubation periods. This is especially relevant in non-endemic countries that is home to an aging demographic of individuals who migrated decades ago from endemic countries. The importance of this concept is emphasized by the persistent neurologic impairment suffered by our case due to untreated type 1 reaction. We also demonstrate the necessity of skin biopsy in distinguishing this diagnosis from other autoimmune mimics in a patient with known autoimmune disease.

## Background

Leprosy, a chronic disease caused by infection by *M. leprae* or *M. lepromatosis,*remains a leading infectious cause of disability worldwide. Leprosy remains a rare diagnosis in Canada, with 24 cases of leprosy identified between 2016 and 2020 [1], typically among immigrants from endemic regions. Leprosy remains an under-recognized diagnosis in non-endemic countries such as Canada, with significant diagnostic delays and resultant neurologic impairment among newly identified cases [2].

The true prevalence of Canada remains unclear and the incidence, if any, of locally-acquired infection is unknown. In the past decade there have been three case reports of leprosy among Canadian-born individuals; while one individual (with *M. lepromatosis*) had no clearly implicated travel history [3], the other two cases involved travel to the Southern United States and Mexico, with genotyping results consistent with *M. leprae,*suggesting zoonotic transmission from armadillos in these same regions where zoonotic transmission is well-described [4–6].

Here we describe a case of *M. leprae* infection manifesting as borderline leprosy with immunologic reaction in an 84-year-old man who had emigrated from Greece to Canada 56 years prior to his symptom onset. Informed consent (written and verbal) was obtained from the patient for the publication of this case and clinical images.

## Case presentation

**Fig. 1:**
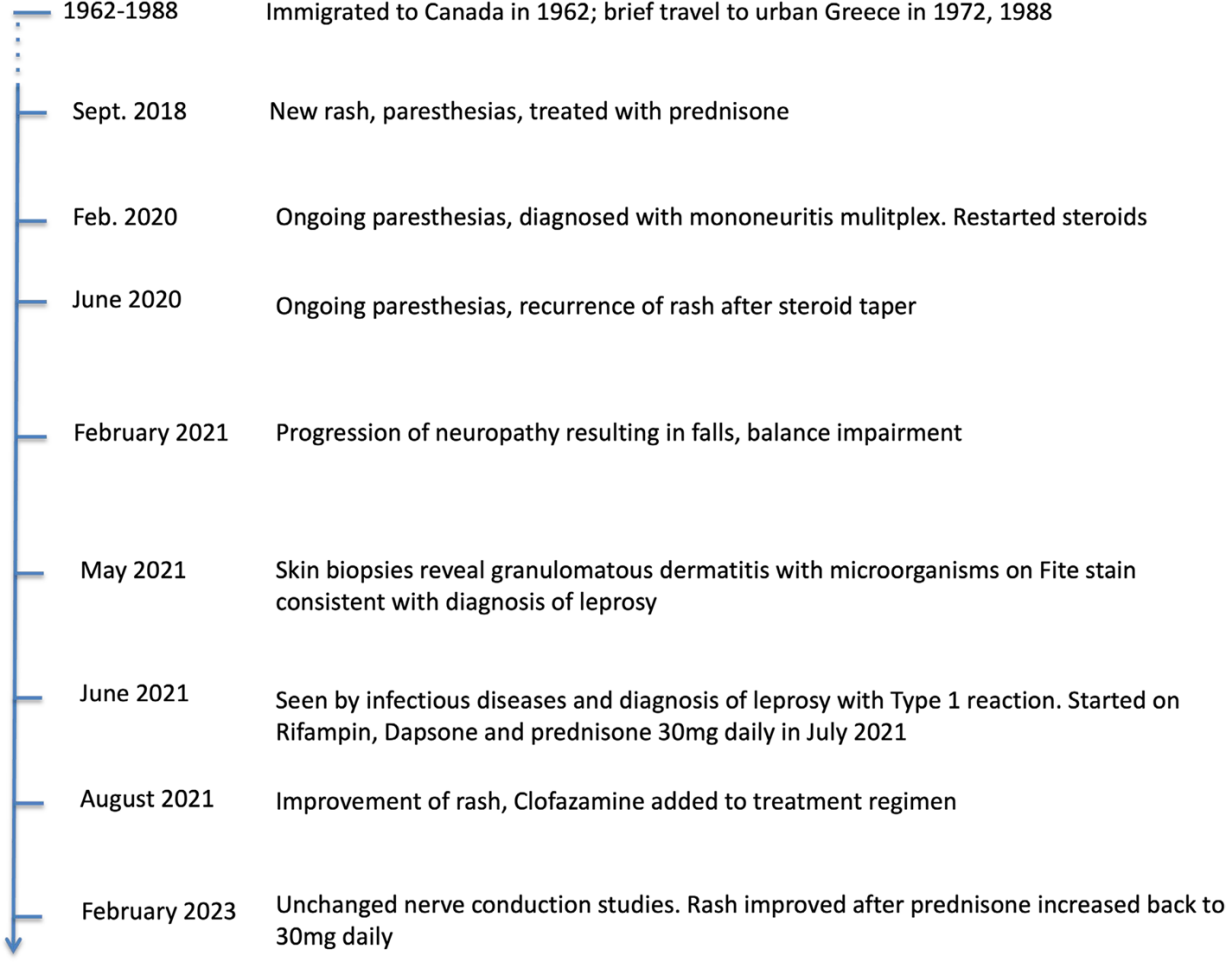
Overview of case timeline

An 84-year old man who immigrated to Canada from Greece in 1962, presented to rheumatology clinic in January 2019 with a 4-month history of paresthesias and leg rash (see Fig. 1 for overview of case timeline). He was followed for rheumatoid arthritis manifesting as erosive arthritis of his wrists and fingers, which was well-controlled with administration of methotrexate and hydroxychloroquine since 2014. His past medical history also included resolved hepatitis B infection, benign hypertension, sleep apnea and COPD.

**Fig. 2:**
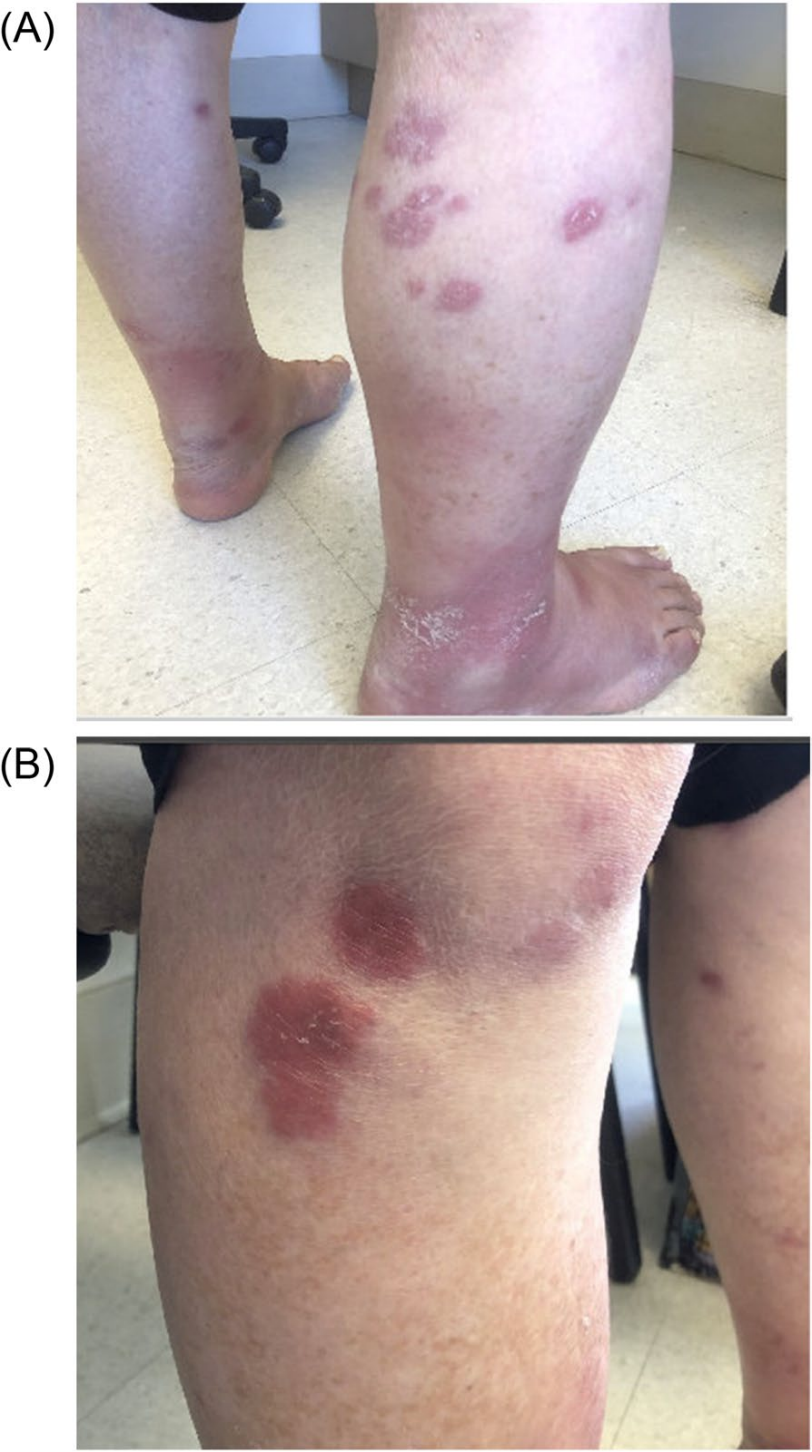
(April 2019) Initial skin lesions prior to steroid therapy

**Fig. 3:**
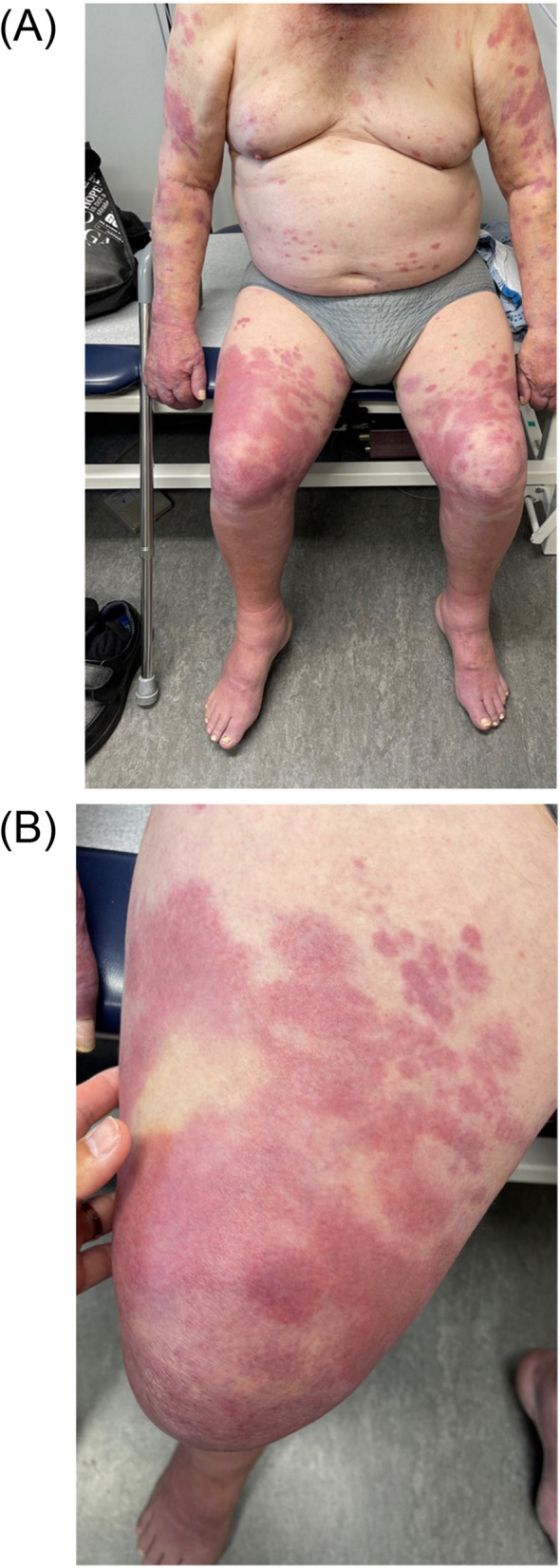
(May 2022) Progression of skin lesions with blanching erythema consistent with Type 1 reaction. **A** Diffuse erythematous skin rash consistent with Type 1 reaction. **B** Erythema was blanching with pressure

When seen in January 2019 he described a 4-month history of rash involving both lower extremities as pruritic dark red annular plaques, (Fig. 2) as well as paresthesias and radiating pain from his shins to the plantar surfaces. By May 2019 his paresthesias progressed to involve his left 1^st^-4^th^ fingers with hypoesthesia (Fig. 3)*.* He was administered with prednisone 30 mg daily for a presumed diagnosis of Sweet syndrome versus atypical erythema nodosum. His rash later resolved and prednisone was discontinued with a tapering course.

In February 2020 he continued to have paresthesias of his lower extremities and fingers of both hands. Nerve conduction studies conducted at that time revealed severe sensorimotor axonal polyneuropathy with asymmetric features, involving both legs, ulnar neuropathies (right greater than left), right radial and median sensory nerves, consistent with mononeuritis multiplex. These symptoms were attributed to rheumatoid arthritis-associated vasculitis and he was started on Prednisone 30 mg daily with sulfasalazine. Sural nerve biopsy was deferred out of concern for delayed wound healing in the context of his concurrent venous stasis.

He had minimal improvement with therapy and by June 2020, after having tapered to 15 mg of prednisone, he developed recurrence of a rash similar in appearance to his earlier lesions, with ovoid, violaceous to pink, non-pruritic, blanchable plaques with some fine scale at his proximal lower extremities bilaterally. Considering the symptoms as manifestation of drug reaction, sulfasalazine was discontinued. A therapeutic trial of tofacitinib was initiated for presumed vasculitic neuropathy and was later discontinued for lack of effect, and his neuropathies were managed symptomatically with pregabalin.

**Fig. 4:**
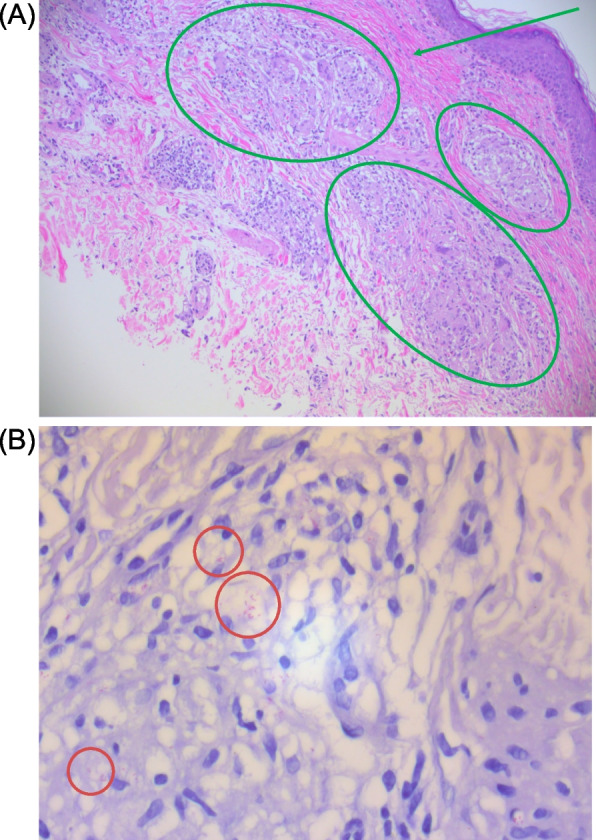
(May 2022): Shave biopsies of left arm. **A** Left arm, shave biopsy, 100 × magnification, H&E. Non-necrotizing granulomas (green ovals) are present in the superficial dermis, associated with some fibrosis (green arrow). **B** Left arm, shave biopsy, 600 × magnification, Fite stain. Acid fast bacilli are present within the histiocytes and granulomas (best seen within the red circles)

His rash further progressed by December 2020, at which point systemic steroids had been discontinued and his lesions were treated with topical steroids alone. By February 2022, he had further progression of neuropathies leading to falls and balance impairment, as well as continued progression of the rash, now involving his legs, abdomen, arms, and back (Fig. 2). He was examined by dermatology in May and three skin biopsies were taken from his right arm and thigh. Pathological examination demonstrated granulomatous dermatitis, without significant necrosis. Within some granulomas brightly and weakly stained acid-fast bacilli (AFB) were noted with Fite and Ziehl–Neelsen stains respectively (Fig. 4). Granulomas were seen adjacent to lymphatics and deep vessels, however nerve fibres were not well-appreciated on the obtained biopsies.

He was seen urgently by the department of infectious diseases in June 2022. His social history revealed no known contacts with individuals with leprosy or exposures to animals such as armadillos; he had not traveled outside Canada apart from two month-long trips to Greece in 1972 and 1988. On examination he was noted to have development of violaceous plaques with fine scaling and nodular skin thickening involving the arms, trunk, back, neck and face. There was no obvious nerve thickening but his patches were hypoesthetic. He had full eyebrows and no ear lobe thickening. He was diagnosed with borderline leprosy with Type 1 reaction and was treated with a three-drug treatment regimen with dapsone, rifampin and clofazimine as recommended by the World Health Organization [7]. In July 2022, he was treated with daily oral dose of rifampicin (600 mg), dapsone (100 mg) and prednisone (30 mg) for Type 1 reaction, with daily dose of Clofazimine (50 mg) added in August 2022 when supply became available. Repeat skin biopsies performed July 14 2022 were sent to Public Health Ontario Laboratories for PCR to identify *M. leprae* DNA, which was detected. Mycobacterial cultures were negative.

**Fig. 5:**
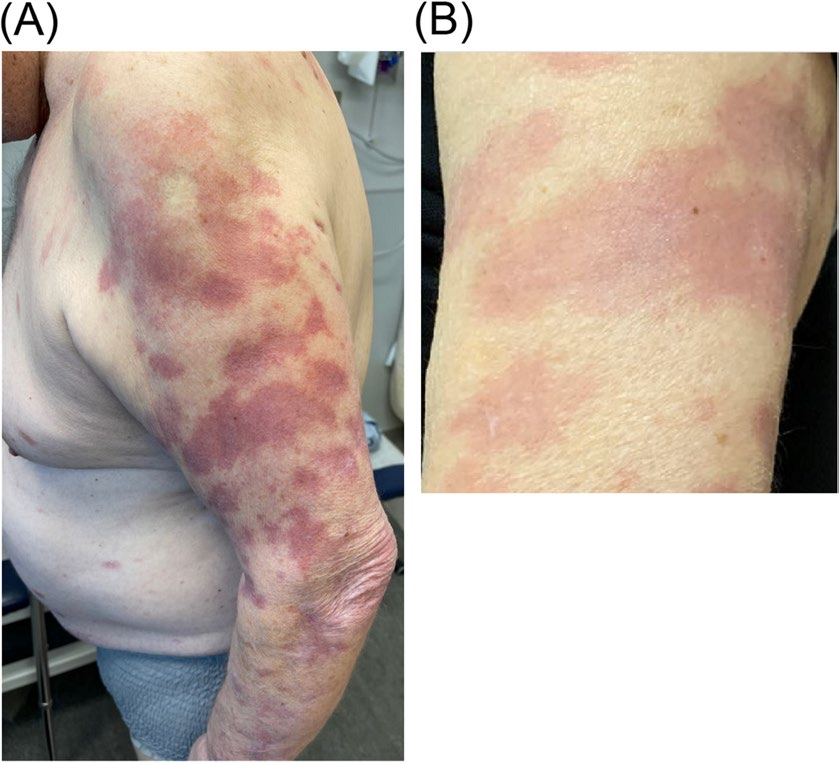
Improvement of erythroderma with antimicrobial and prednisone treatment. Left arm prior to therapy (May 2022) (**A**) versus after antimicrobial and steroid therapy (August 2022) (**B**)

**Fig. 6:**
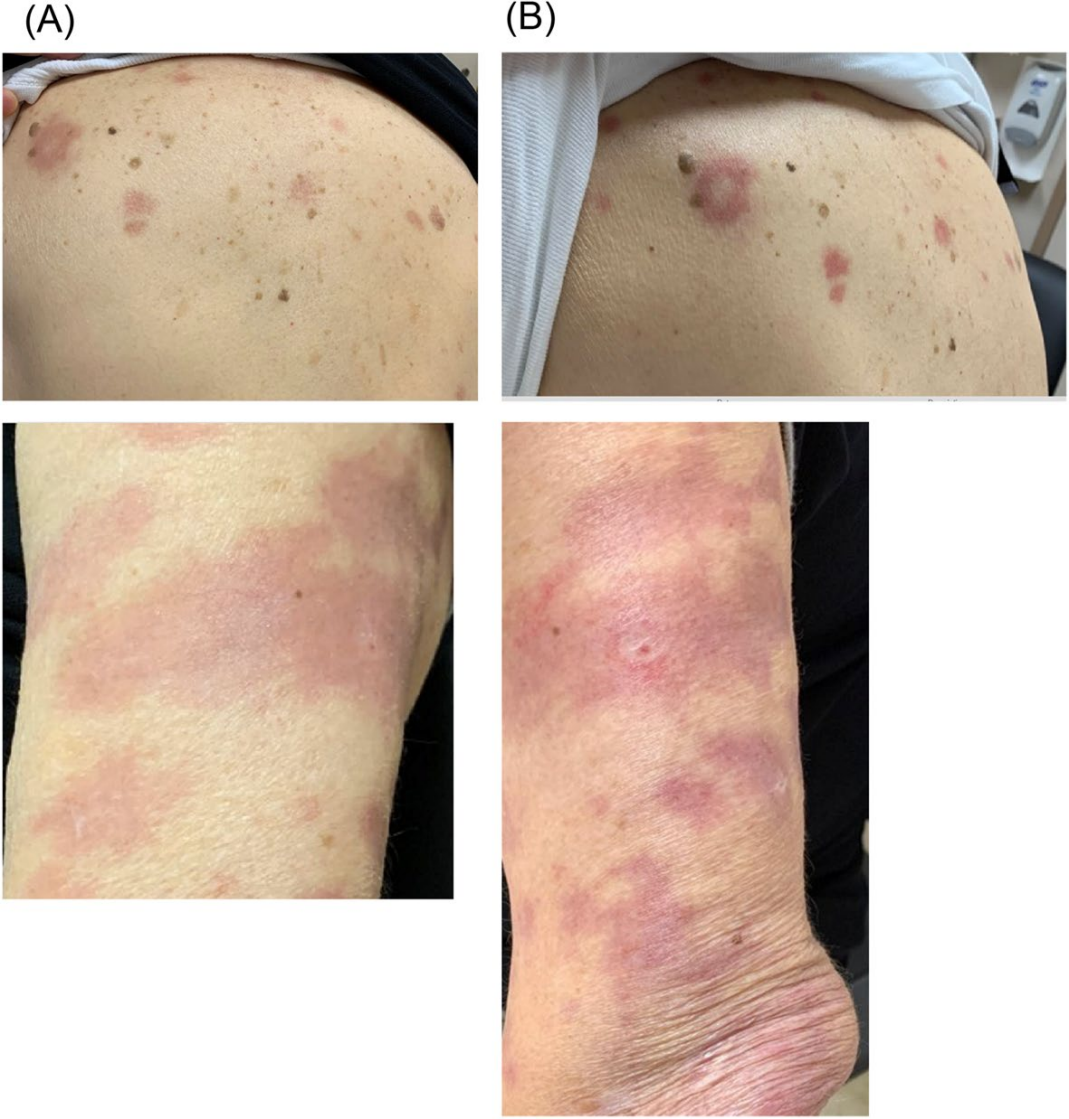
Worsening of erythroderma following tapering of prednisone to less than 10 mg daily. Skin lesions visualized on back and left arm while on prednisone 30 mg daily, August 2022) (**A**) versus after tapering to less than 10 mg daily (October 2022) (**B**)

In August, after nearly two months of therapy including prednisone, the erythema surrounding his skin lesions had significantly improved (Fig. 5). Clofazimine was started at 50 mg PO daily. After tapering to less than 10 mg of Prednisone by October 2022, he had a recurrence of Type 1 reaction (Fig. 6) and his daily oral dose prednisone was again increased to 30 mg. Repeat skin biopsies on November 2022 revealed mild lymphocytic infiltration without any presence of AFB. By February 2023 his paraesthesia subjectively improved, however there was no improvement in sensation on examination and repeat nerve conduction studies remained unchanged. Ultimately, while the erythroderma surrounding his skin lesions had largely resolved, his neurologic deficits were deemed irreversible by the following year, and his prednisone was gradually tapered.

## Discussion and conclusions

Leprosy can result in significant neurologic impairment if left undiagnosed and untreated, particularly in the context of immunologic reaction as in the presented case. This case is atypical given the lack of relevant exposure or travel history, suggesting either a markedly prolonged incubation period or locally acquired infection in Canada. While we are unfortunately not able to use genotyping to determine the regional origin of the infecting *M. leprae* strain, our case illustrates number of important considerations for the evaluation of leprosy in Canada, both from an epidemiological and clinical perspective.

Our case most likely represents a presentation after a markedly prolonged incubation period of as many as 56 years since his immigration from Greece to Canada. To our knowledge, this is the longest incubation period reported to-date. It is less likely that our case had acquired leprosy from his brief travel to endemic regions in Greece, given that autochthonous cases within Greece [8] are rare and typically occur within rural regions, whereas the patient’s travel was limited to urban centres. That our patient developed an imported case of leprosy after a prolonged incubation of 56 years raises important considerations for low-incidence healthcare settings. The World Health Organization has suggested that while the median incubation period is 5 years, onset of symptoms can begin as many as 20 years after infection [9, 10], however longer incubation periods have recently been described including the longest reported incubation period of 50 years [11]. Other low-incidence countries such as Norway have reported an increasing proportion of leprosy cases involving prolonged incubation periods with increasing age of onset [12]. Apart from age-related immunosenescence, the transition from quiescent to symptomatic disease may be influenced by immunosuppressant medication [13], which may have been a factor in our case. As the large demographic of Canadians born in endemic countries reaches the advances of age in the coming years [14], it will become increasingly important to consider prolonged incubation periods of leprosy both from a clinical and public health perspective.

Given that the true prevalence of *M. leprae*infection in Canada is obscured by its low transmissibility and frequently delayed diagnosis in low-incidence settings [15], it is theoretically possible that our case represents a true autochthonous infection within Canada. In the absence of any reported high-risk animal exposures, it is possible that our case acquired *M. leprae* infection due to exposure to an infected individual with imported infection within Canada. However, this is very unlikely. To-date there has only been one case report of reasonable probability of leprosy-like infection acquired within Canada, which involved the related but distinct pathogen, *M. lepromatosis* [3]. This pathogen is less commonly identified compared to *M. leprae*, but has been reported sporadically in non-endemic countries outside of Mexico and Central and South America [16]. While the PCR assay used in our case is highly specific for *M. leprae*, this assay would not have detected co-infection with *M. lepromatosis*, a rare occurrence described in approximately three percent of human *M. leprae*infections [16]. Overall, our case does not necessarily indicate concern for endemic leprosy within Canada. Additional cases of *M. leprae* within Canada lacking obvious pertinent exposures may suggest a need to reassess the local epidemiology of this infection.

Type 1 leprosy reaction, which is a cell-mediated immune response directed towards bacilli within Schwann cells and the dermis [17], is considered a medical emergency requiring early diagnosis and treatment to prevent irreversible neurological impairment. The diagnostic delay in our case highlights important lessons for discerning leprosy and leprosy reactions from the manifestations of rheumatoid arthritis, which is a far more common diagnosis particularly in non-endemic settings such as Canada. First, leprosy can occasionally be misdiagnosed as rheumatoid arthritis as it can manifest as inflammatory polyarthritis of the small joints with positive rheumatoid factor [18]. While our patient’s erosive disease and anti-CCP serology are more specific for rheumatoid arthritis [18], this diagnosis clouded the diagnosis of his mononeuritis multiplex as a Type 1 leprosy reaction. Second, treatment directed towards rheumatoid arthritis and autoimmune vasculitis may partially treat leprosy reactions, which can mask the diagnosis and delay definitive treatment, as in our case [19]. Our case emphasizes the importance of considering leprosy and obtaining biopsies early in the evaluation of the patient with presumed inflammatory neuropathy, particularly in cases with associated demographic risk factors or skin findings, or without the expected response to treatment directed towards rheumatologic disease.

Within the Ridley-Jopling classification system, borderline leprosy denotes the disease state that exists between the two poles in the clinical spectrum of disease. Between tuberculoid leprosy (robust cell-mediated immunity with mild disease) and lepromatous leprosy (impaired immunity with high bacillary load), borderline leprosy represents an unstable transitional state in which cell-mediated immunity may shift to pole [20]. Because leprosy reactions result from these immunologic transitions, borderline leprosy accounts for the majority of reactions and overall burden of disease worldwide [20]. Therefore, early recognition of the variable clinical features of this disease state is particularly important.

Certain limitations should be acknowledged. First, it is possible that mild symptoms may not have been reported and the true incubation period was shorter than described, however this is less likely given the thorough and regular documentation throughout this patient’s course of treatment. Second, the lack of visualized nerve fibres on pathology prevents us from attributing his neuropathy to leprosy and immunologic reaction with absolute certainty, although his clinical improvement and overall presentation is strongly compatible with this diagnosis. Finally, while the patient endorsed adherence to therapy as prescribed, this could not be verified in our study.

Our case serves to demonstrate the importance of early consideration and diagnosis of leprosy in cases with a fitting clinical presentation, even with unusually prolonged incubation periods or in the absence of obvious epidemiologic risk factors for infection. This is of particular importance in the context of immunologic reaction, which represent a medical emergency that risks irreversible neurologic damage. This is especially relevant while evaluating patients who immigrated from endemic regions in the remote past, which is an increasing demographic in many non-endemic settings. Our case also demonstrates the utility of biopsy to confidently distinguish this diagnosis from autoimmune mimics. Further research is required to more clearly define the epidemiology of leprosy in Canada and other low-incidence settings, including overall prevalence, and the predictors and time course of the transition from quiescent to symptomatic leprosy.

## Data Availability

Not applicable.
